# Comparison of time consumption and success rate between CT angiography- and CT perfusion- based imaging assessment strategy for the patients with acute ischemic stroke

**DOI:** 10.1186/s12880-022-00880-9

**Published:** 2022-08-29

**Authors:** Yue Chu, Gao Ma, Xiao-Quan Xu, Shan-Shan Lu, Hai-Bin Shi, Sheng Liu, Qiang-Hui Liu, Fei-Yun Wu

**Affiliations:** 1 Department of Radiology, First Affiliated Hospital of Nanjing Medical University, No. 300, Guangzhou Rd., Nanjing, China; 2 Department of Interventional Radiology, First Affiliated Hospital of Nanjing Medical University, Nanjing, China; 3 Department of Emergency, First Affiliated Hospital of Nanjing Medical University, Nanjing, China

**Keywords:** Acute ischemic stroke, Perfusion, Angiography, Time consumption, Success rate

## Abstract

**Background:**

Our study aimed to compare the time consumption and success rate between CTA- and CTP- based assessment strategy, and to clarify the risk factors associated with the CTP scan failure.

**Methods:**

Clinical and radiological data of 437 consecutive AIS patients who underwent multiphase CTA or CTP for pre-treatment evaluation were retrospectively enrolled (CTA group, n = 302; CTP group, n = 135). Time consumption and success rate of CTA- and CTP- based assessment strategy were compared using Mann–Whitney U test and Chi-Squared Test. Univariate analysis and receiver operating curve analysis were used to clarify the risk factors, and their performance in predicting the CTP scan failure.

**Results:**

Time consumption of CTP scan and reconstruction was significantly longer than that of CTA [775 s vs 263.5 s, *P* < 0.001]. CTP scan showed significantly higher failure rate than CTA (11% vs 1%, *P* < 0.001). Severe motion was the most common cause of CTP failure (n = 12, 80%). Baseline National Institute of Health Stroke Scale (NIHSS) score in CTP failure group was significantly higher than that in CTP success group [17 vs 13, *P* = 0.007]. Baseline NIHSS score of 11 was the optimal threshold value to predict CTP failure with an area under the curve of 0.715, a sensitivity of 86.7%, and a specificity of 45.0%.

**Conclusions:**

CTP- based strategy showed longer time consumption and higher failure rate than CTA- based strategy. High baseline NIHSS score was significantly associated with CTP scan failure in AIS patients.

## Background

Stroke is a leading cause of disability and mortality in the worldwide [1]. Multimodal computed tomography (CT), including non-enhanced CT (NCCT), CT angiography (CTA), CT perfusion (CTP) imaging, are important imaging techniques for accurately assessing the patients with acute ischemic stroke (AIS) before treatment [2]. In clinical practice, NCCT is mainly used to exclude the intracerebral hemorrhage, and estimate the extent of early ischemic injury using the Alberta Stroke Program Early CT Score (ASPECTS) [3]. CTA can help to identify whether large vessel occlusion (LVO) occurred or not, and grade the collateral flow status [4, 5]. CTP can accurately quantify the “tissue window” including the volume of infarct core and ischemic penumbra, so as to establish a reasonable treatment strategy for the AIS patients, especially in an extended time window [6, 7]. However, how to establish the most reasonable imaging assessment strategy for the AIS patients in different time window is still in debate.

The 2019 AHA/ASA guideline recommended CTP as the pre-treatment imaging assessment for AIS patients with an onset time over 6 h according to DEFUSE 3 trial [2, 7]. While for AIS patients within 6 h of onset with LVO, since the time to reperfusion has a significant effect on clinical outcome, the 2019 AHA/ASA guideline recommended angiography assessment only for selecting mechanical thrombectomy (MT) eligibility, and treatment should not be delayed by additional perfusion imaging [2]. Previous studies reported that CTP needed more time to acquire, process and interpret, and hold high rate of post-processing failure. These disadvantages might contribute to the recommendation not to use CTP for imaging assessment for patients within the time window [8, 9]. However, Olivot et al. recently reported that twenty-nine percents of the AIS patients with onset time less than 6 h had no target mismatch. Using CTP to evaluate target mismatch for patients within the time window was also important for identifying patients had potential to respond favorably to reperfusion therapy [10]. Therefore, there was still no consensus on how to establish the imaging assessing strategy for the AIS patients in different time window. In our opinion, full understanding of the time usage and failure rate of CTA- and CTP- based imaging assessment strategies was of great importance for making the most reasonable imaging protocol for AIS patients.

Therefore, the purpose of our study was to compare the time usage and success rate between CTA- and CTP- based imaging assessment strategy for the AIS patients, and to clarify the risk factors potentially associated with CTP scan failure.

## Materials and methods

### Patient selection

The study was approved by our center’s ethical committee for studies in humans. The requirement for a written informed consent was waived due to the retrospective nature of the study. We included consecutive patients admitted to our emergency department with a diagnosis of AIS from March 2018 to October 2019. We selected patients who (1) were older than 18 years; (2) presented to the emergency department with symptoms consistent with AIS; (3) underwent NCCT + CTA or NCCT + CTP for pre-treatment evaluation; (4) were treated by endovascular treatment (EVT) or intravenous thrombolysis (IVT).

### Image protocol

Baseline CT was performed using a 128-section CT scanner (Optima CT 660, GE Medical Systems). For AIS patients with an onset time less than 5 h, imaging protocol included NCCT (120 kVp, 100–350 auto-mAs, and contiguous 5-mm axial sections ranging from the vertex to the skull base) and multiphase CTA (mCTA). The mCTA generated time-resolved cerebral angiograms of brain vasculature from the skull base to the vertex in three phases after contrast agent injection. Aortic arch vertex CT angiography made up the first phase. Image acquisition was timed to occur during the peak arterial phase in the healthy brain and was triggered by bolus monitoring. The remaining two phases are from the skull base to the vertex in the equilibrium/peak venous and late venous phases in the healthy brain. Images were acquired with a 0.625-mm section thickness. The second phase was acquired after a delay of 8 s that allows for table repositioning to the skull base. Scanning duration for the first phase was 5.1 s and for each additional phase was 2.6 s. A total of 50-mL nonionic iodinated contrast (iopromide, Ultravist 370, Bayer Schering Pharma) was administered at a flow rate of 4 mL/s, followed by a 40-mL saline chaser at a rate of 4 mL/s. The CT dose indexes (CTDI_vol_) were 8.14 mGy, 5.70 mGy, and 5.71 mGy for mCTA scan in three phases, respectively. The average total dose-length products (DLP) were 326.78 ± 0.10 mGy*cm, 117.63 ± 0.07 mGy*cm, and 117.73 ± 0.05 mGy*cm, respectively.

For AIS patients in whom the onset time ≥ 5 h or with unclear time, imaging protocol included NCCT and CTP. CTP scan was conducted using the cradle table technique, allowing a coverage of whole brain. The imaging parameters for CTP scan were as follows: 4-dimensional adaptive spiral mode, periodic spiral approach, 80 mm in z-axis, 1.7 s temporal resolution, 30 consecutive spiral scans, 100 kVp, 200 mAs, rotation time of 0.4 s and 0.984 maximum pitch. A scan delay of 2 s was applied after injecting 50-mL (flow rate 5 mL/s) nonionic iodinated contrast (iopromide, Ultravist 370, Bayer Schering Pharma, Germany), followed by a 30-mL saline at the same rate. As a standard scan model, the CTDI_vol_ was 315.68 mmGy, and the total DLP was 2998.96 mmGy*cm for each CTP scan. CTP data were analyzed retrospectively in a commercial software (Advantage Workstation 4.7; GE Healthcare, Milwaukee, WI, USA), using singular value deconvolution algorithms. Simulated CTA source image was reconstructed from the peak arterial phase in the normal distal ICA of the source CTP images for detecting vessel occlusion, based on the arterial input function (AIF). CTP data were reconstructed with a thickness of 5 mm for perfusion analysis, and with a slice thickness of 0.625 mm for CTA analysis.

### Data analysis

The patients were divided into CTA group and CTP group based on the imaging modality used. We collected and recorded the time consumption of NCCT scan (defined as time interval from the first location image to the last NCCT image), CTA or CTP scan and reconstruction (defined as time interval from the last NCCT image to the termination of CTA or CTP reconstruction), and the total imaging duration (NCCT + CTA or CTP time usage). All the time information was retrospective obtained from the picture archiving and communication system equipped in our stoke center (Version 12.1, Carestream Vue, Carestream Health). In CTA scan, failure was defined as poor visualization of vascular structure which would influence subsequent imaging evaluation. For CTP scan, failure was defined as abnormal perfusion curves. If perfusion curve showed as a typical wash-in and wash-out pattern, we would define the CTP scan as successful. If the perfusion curve was chaotic or wash-out phases were not finished, we would define it as a failure. The potential causes of CTP or CTA scan failure included severe motion, streak artifact, and poor arrival of contrast bolus due to poor basic condition of vessel. Two raters (both with more than 5 years experience in neuroradiology) who were blinded to the clinical data assessed whether failures occurred or not. In case of disagreement between two raters, consensus was reached by discussion with a senior radiologist (with more than 20 years experience in neuroradiology). Demographic, clinical and imaging information such as age, gender, baseline National Institute of Health Stroke Scale (NIHSS) score, onset time and occlusion site were collected for univariate analysis between CTP success group and failure group.

### Statistical analysis

Continuous variables were reported as median with interquartile range (IQR). Categorical variables were reported as proportions. The comparison of time duration between CTA group and CTP group was used Mann–Whitney U test. Chi-Squared test was used to compare the failure rates between CTA group and CTP group. Demographic, clinical and imaging information between CTP success and failure group were compared using Chi-Squared Test or Mann–Whitney U test. Receiver operating characteristic (ROC) curves were used to assess the ability of the significant associated factors to predict the CTP scan failures. Area under the ROC curve (AUC), sensitivity and specificity were calculated and reported. All statistical analyses were performed using MedCalc (version 12.3.0) or SPSS (version 23.0). A two-sided P value less than 0.05 was considered significant.

## Result

### Patients

Among 437 patients enrolled in our study, 302 (69%) patients underwent CTA- based, while 135 (31%) patents underwent CTP- based imaging assessment strategy. The mean age of the patients in CTA group was 70 (IQR 61–77) years, and 60% were male. The median age of the patients was 67 (IQR 60–75) in CTP group, and 61% were male. The median baseline NIHSS score was 12 (IQR 7–19) for CTA group, and 13 (IQR 9–17) for CTP group. The median time interval from stroke onset to CT scan was 2.5 (IQR 1.5–3.5) hours for CTA group, and 6 (IQR 5–8.5) hours for patients in CTP group with clear onset time. Among them 32 (24%) patients in CTP group had awakened from sleep with symptoms of a stroke and with unclear onset time, and the onset time was the time they were last seen well. The number of patients with occlusion in internal carotid artery or MCA, basilar and other sites was 206 (68%), 21 (7%) and 75 (25%) respectively for the CTA group, while 97 (72%), 10 (7%) and 28 (21%) for the CTP group. In the CTA group, 61 (20%) patients were treated with EVT after intravenous tPA, 152 (50%) patients were treated with intravenous tPA alone, and 89 (30%) patients were treated with EVT alone. While in the CTP group, 16 (12%) patients received EVT after intravenous tPA, 32 (24%) patients received intravenous tPA alone, and 87 (64%) patients received EVT alone. Baseline demographic and clinical characteristics of CTA and CTP group are summarized in Table 1.

**Table 1:** Demographic and clinical characteristics of patients in CTA and CTP group

Variables [Median (IQR) or N (%)]	CTA group (N = 302)	CTP group (N = 135)
Age, years	70 (61–77)	67 (60–75)
Sex, male	181 (60%)	82 (61%)
Baseline NIHSS	12 (7–19)	13 (9–17)
Time interval from onset to imaging, hours	2.5 (1.5–3.5)	6 (5–8.5)
Patients awakened with stroke symptoms	0 (0%)	32 (24%)
Treatment		
-Intravenous tPA	152 (50%)	32 (24%)
-EVT	89 (30%)	87 (64%)
-Intravenous tPA + EVT	61 (20%)	16 (12%)
Occlusion site		
-ICA or MCA	206 (68%)	97 (72%)
-Basilar	21 (7%)	10 (7%)
-Other site	75 (25%)	28 (21%)

CTA indicates computed tomography angiography; CTP, computed tomography perfusion; NIHSS, National Institutes of Health Stroke Scale; IQR, interquartile range; SD, standard deviation; MCA, middle cerebral artery; ICA, internal carotid artery

### Comparison of time usage

**Fig. 1:**
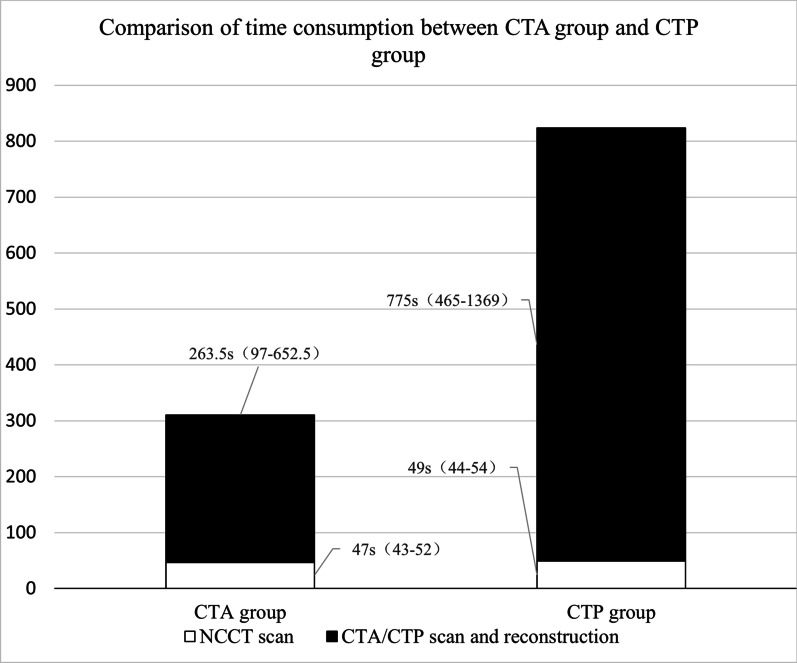
Comparison of time consumption between CTA and CTP group. CTA indicates computed tomography angiography; CTP, computed tomography perfusion; IQR, interquartile range

Comparison of time usage between CTA and CTP group was shown in Table 2 and Fig. 1. There was no significant difference in the time usage of NCCT can between CTA and CTP group [IQR, 47 (43–52) sec vs 49 (44–54) sec, *P* = 0.055]. However, CTP needed significantly longer time for scan and imaging reconstruction than CTA [IQR, 263.5 (97–652.5) sec vs 775 (465–1369) sec, *P* < 0.001]. Total time duration for NCCT + CTP assessment strategy was significantly longer than that of NCCT + CTA strategy [828 (503–1413) sec vs 312.5 (148–711) sec, *P* < 0.001].

**Table 2:** Comparison of time consumption between CTA and CTP group

Variables [Median (IQR)]	Time of NCCT scan	Time of CTA/CTP scan and reconstruction	Total time consumption
CTA group, seconds	47 (43–52)	263.5 (97–652.5)	312.5 (148–711)
CTP group, seconds	49 (44–54)	775 (465–1369)	828 (503–1413)
P value	0.055	< 0.001	< 0.001

CTA indicates computed tomography angiography; CTP, computed tomography perfusion; IQR, interquartile range

### Comparison of scan failure rate and the factors leading to scan failure

**Fig. 2:**
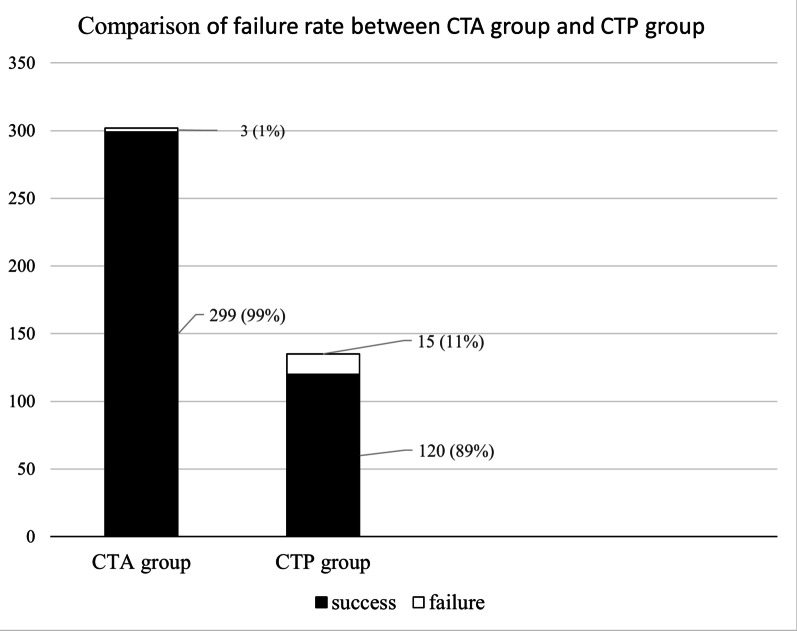
Comparison of failure rate between CTA and CTP group. CTA indicates computed tomography angiography; CTP, computed tomography perfusion; IQR, interquartile range

The scan failure rate of CTP group was 11% (15/135), while that of CTA group was 1% (3/302). The scan failure rate of CTP group was significantly higher than CTA group (*P* < 0.001). The comparison of failure rate between CTA and CTP group was shown in Fig. 2. Causes of CTP scan failure included severe motion (n = 12, 80%), streak artifact (n = 1, 7%) and poor arrival of contrast (n = 2, 13%), while causes of CTA scan failure were severe motion (n = 2, 67%) and streak artifact (n = 1, 33%). The comparisons of demographic, clinical and imaging variables between CTP failure and CTP success group were shown in Table 3. According to the univariate analysis results, baseline NIHSS score was the only variable associated with CTP scan failure [IQR, 17 (13–20) vs 13 (8–16), P = 0.007].

**Table 3:** Characteristics and differences between patients with CTP failure and CTP success

Variables [Median (IQR) or N (%)]	CTP Failure (N = 15)	CTP Success (N = 120)	*P* value
Age, years	75 (56–79)	67 (60–74)	0.445
Sex, male	7 (47%)	76 (63%)	0.211
Baseline NIHSS	17 (13–20)	13 (8–16)	**0.007**
Time from symptom onset to CTP, hours	6 (5–8.5)	7 (5–9)	0.633
Treatment			0.322
-Intravenous tPA	4 (27%)	28 (23%)	
-EVT	11 (73%)	76 (63%)	
-Intravenous tPA + EVT	0 (0%)	16 (14%)	
Occlusion site			0.735
-ICA or MCA	12 (80%)	85 (71%)	
-Basilar	1 (7%)	9 (7%)	
-Other site	2 (13%)	26 (22%)	

A two-side *P* value less than 0.05 was considered significant and was given in bold

CTP indicates computed tomography perfusion; NIHSS, National Institutes of Health Stroke Scale; IQR, interquartile range; tPA, tissue plasminogen activator; EVT, endovascular treatment; MCA, middle cerebral artery; ICA, internal carotid artery

**Fig. 3:**
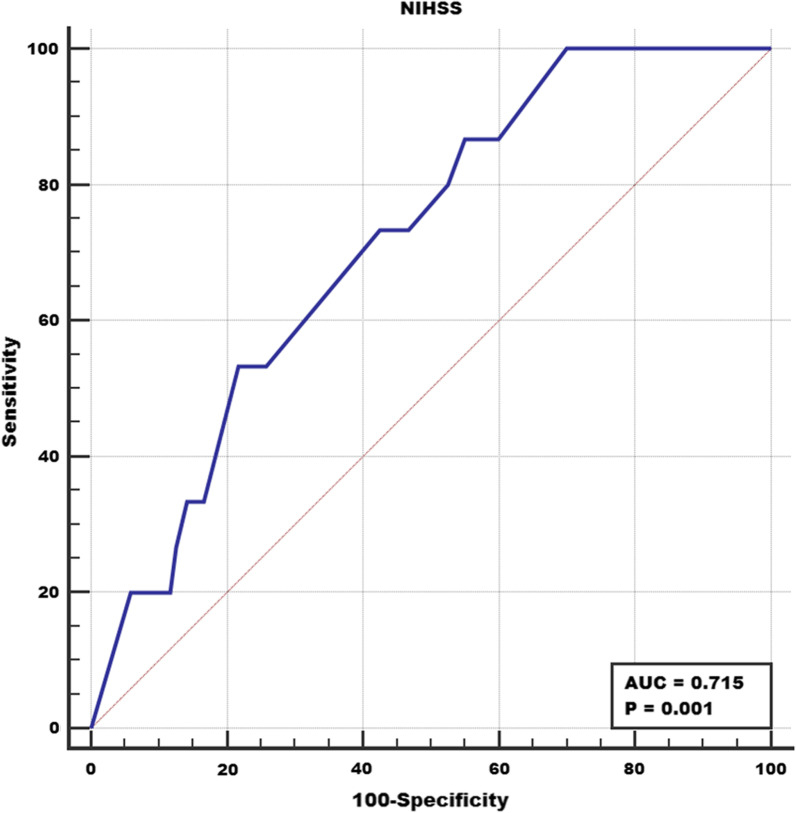
Receiver-operating characteristic curves of using baseline National Institutes of Health Stroke Scale to predict the failure of CTP

ROC curves of using baseline NIHSS score to predict CTP scan failure were shown in Fig. 3. Using a baseline NIHSS score of 11 as the threshold value, optimal performance could be achieved for predicting CTP scan failure (AUC, 0.715; sensitivity, 86.7%; specificity, 45.0%).

## Discussion

Our study demonstrated three major findings. First, there was no significant difference in the time consumption for NCCT scan between CTA- and CTP- based imaging assessment strategy. However, CTP- based imaging assessment strategy needed significant longer time for imaging scan and reconstruction than CTA- based strategy. The time delayed for CTP- based strategy was approximately 10 min. Second, we found that CTP scan showed a higher failure rate than CTA scan. The failure rate of CTP scan was 11%, and motion was the most common cause. Third, we found the admission NIHSS were significantly associated with CTP scan failure.

Currently, CTP and CTA were two main imaging techniques commonly used for assessing the patients with AIS. Besides differential diagnosing stroke mimics and improving the detection rate of medium vessel occlusion [11], CTP could provide an objective and quantitative assessment of the volume of ischemic core and penumbra, and subsequently had been wide used to selected patients with anterior LVO in both early and extended window for EVT [7, 10, 12]. However, a relative longer time consumption which might potentially result in reperfusion delay and relative larger dose of radiation for CTP scan must be taken into consideration [8]. The main role of CTA scan was to clarify whether a LVO existed or not. Beside the well-known advantage of speediness, several researchers recently reported that there was close relationship between CTP- derived and CTA- derived diagnostic information, especially when mCTA used [13, 14].

In our study, CTP- based imaging assessment strategy showed relatively longer time consumption than CTA- based strategy. Multiphase CTA included a three-time phases scan, while CTP scan contained 30 consecutive spiral scans of the brain. More images raw data would be obtained from CTP scan, and subsequent transform of raw data from scan workstation to reconstruction workstation might be one reason of time delay in CTP- based strategy. Besides that, compared with reconstruction of vascular image from CTA, reconstruction of CTP parametric maps seemed to be a more complicated process. This situation might also be associated the time delay of CTP- based strategy. Considering the potential association between the longer time delay and the poorer clinical outcome, effectively shortening the time usage of CTP- based strategy had important clinical significance. Recently, increasing large comprehensive stroke centers have applied the automated perfusion analysis software to process the perfusion data. It may dramatically shorten the time usage of CTP reconstruction and subsequent analysis [12, 15, 16]. While in primary stroke center, the automated reconstruction software was still not popularized. The decision to open up an advanced CTP examination to assess the AIS patients should be taken into careful consideration for the potential longer time consumption of CTP imaging strategy.

Besides longer time consumption, CTP also showed higher failure rate than CTA in our study. Kauw et al. reported a CTP failure rate of 11%, and they found that motion was the leading cause, followed by streak artifacts and poor contrast bolus arrival, which was in line with our study [9]. The main reason for the higher failure rate of CTP might be that it was difficult for the patients to remain stationary during the relatively longer scan time. In consistent with the prior study, we found that baseline NIHSS was the risk factor that significantly associated with CTP failure [9]. Patients with higher baseline NIHSS would be neurologically more severe, more restless, and more likely to be mobile in the scanning process. We recommended to take caution on the patients with high NIHSS score, and to take effective measures such as fixed band and sedation drugs to keep them static during CTP scan. According to our result, we would recommend the sedative medication before CT scan for the stroke patients with a NIHSS higher than 11. Certainly, we admitted that the optimal threshold value of NIHSS should be derived based on further study with larger sample size.

When CTP scan failure occurred, repeated CTP scan was usually not suggested because of that waiting the clearance of the contrast retention within the brain would increase the time consumption. Therefore, some previous studies tried to find fungible imaging biomarkers. Nannoni et al. reported that NCCT- based ASPECTS correlated significantly with the CTP infarct core, especially in the AIS patients with late window and large-vessel occlusion. Therefore, they concluded that the use of ASPECTS could be a surrogate maker of CTP core in late-arriving AIS patients with large-vessel occlusion [14, 17]. In addition, simulated mCTA reconstructed from CTP raw data was also reported to be useful in reflecting the perfusion information to a certain extent. Better collateral circulation was tightly correlated with small ischemic core volumes. A mCTA collateral score more than 3 could optimally predict a target mismatch on CTP and a good clinical outcome in AIS patients [13].

Our study had some limitations. First, our study was a retrospective study conducted in single center, and with relatively small sample size. Considering different imaging modalities and different post-processing software were used in different centers, further multi-center trials with more study population were needed to be performed to confirm the reliability of our results. Second, the time duration of CTA and CTP post-processing could be influenced by the experience of different radiologists, therefore a unified post-processing train standard should be taken in further studies. Last but not least, time data was retrospectively recorded from the picture archiving and communication system. By contrast, the time information obtained in an informationized approach might be more accurate.

## Conclusion

In summary, our study found that CTP- based imaging assessment strategy needed more time consumption, and showed higher failure rate than CTA- based strategy. High baseline NIHSS score might be a risk factor associated with the CTP scan failure in AIS patients.

## Data Availability

The data that support the findings of this study are available from the corresponding author, Fei-Yun Wu, upon reasonable request.
